# Reliability and usefulness of the single leg heel raise balance test in patients with chronic ankle instability

**DOI:** 10.1038/s41598-021-99466-8

**Published:** 2021-10-13

**Authors:** Jin Hyuck Lee, Hae Woon Jung, Taek Sung Jung, Woo Young Jang

**Affiliations:** 1 Department of Sports Medical Center, Korea University College of Medicine Anam Hospital, Seoul, Korea; 2 Department of Orthopedic Surgery, College of Medicine, Korea University, 73, Inchon‑ro, Seongbuk‑gu, Seoul, 02841 Republic of Korea; 3 Department of Pediatrics, Kyung Hee University Medical Center, Seoul, South Korea; 4 TMX Limited, Seoul, Republic of Korea

**Keywords:** Health care, Medical research

## Abstract

We aimed to analyze the differences in static (including conventional and modified [single-leg heel-raise balance]) and dynamic postural stability and muscle endurance between patients with chronic ankle instability (CAI) and healthy controls, and to determine the reliability and usefulness of the single-leg heel-raise balance test in patients with CAI. In total, 26 patients with CAI and 26 healthy controls were enrolled. Postural stability was assessed using a postural stabilometry system. Muscle endurance was measured in dorsiflexion and plantarflexion using an isokinetic device. Modified static postural stability (*P* < 0.001) and dynamic postural stability (*P* < 0.001) were significantly poorer in the affected ankles of patients with CAI than in the controls. Plantarflexion endurance was significantly lower in the affected ankles of the patients with CAI than in the controls (*P* = 0.023). Modified static postural stability significantly correlated with plantarflexion endurance in both groups (CAI group: r = − 0.470, *P* = 0.015; healthy controls group: r = − 0.413, *P* = 0.036). Plantarflexion endurance was a significant risk factor for modified static postural stability in both the CAI group (R^2^ = 0.221, *P* = 0.015) and healthy controls (R^2^ = 0.170, *P* = 0.036). Given the reliability of the modified static postural stability test, clinicians and therapists should consider using it to assess improvements in postural stability and muscle endurance in patients with CAI before and after rehabilitation.

## Introduction

Ankle sprains are commonly experienced during sports activities, and lateral ankle sprain occurs with a particularly high frequency^1,2^. Lateral ankle sprain can cause chronic ankle instability (CAI)^3,4^ due to ligamentous complex injury, muscle weakness, and lack of proprioception and neuromuscular control^5,6^. Therefore, clinicians and therapists recommend specific rehabilitation to recover strength, proprioception, and neuromuscular control in patients with CAI^4^.

As most previous studies have reported a lack of proprioception in patients with CAI^4,5,7–10^, the single-leg balance test has been used to determine the prognosis of functional recovery, including proprioception, before and after treatment such as surgery and conservative rehabilitation in patients with CAI. However, this balance test comprises static and dynamic postural stability tests^8,11,12^. It has been reported that static^12,13^ and dynamic^8,12–14^ postural stability deficits are the most important factors to consider in the treatment of patients with CAI. Furthermore, a previous study reported that calf muscle endurance was lower in patients with CAI who underwent rehabilitation than in those with CAI who underwent the modified Broström procedure^9^; thus, it is an important factor in the treatment of these patients. To our knowledge, only one previous study has evaluated calf muscle strength using the heel-raise test in patients with CAI^15^. The heel-raise test is commonly used to assess calf muscle endurance^16–19^ and is cost-effective and highly reliable^18^. However, to our knowledge, no studies have yet examined the single-leg heel-raise balance test (modified static postural stability test) as a combination of the single-leg balance and heel-raise tests in patients with CAI and healthy controls. In particular, this test may be considered an important evaluation method to confirm the existing problems of patients with CAI because it can simultaneously evaluate the single-leg balance ability and calf muscle endurance. Moreover, whether the modified static postural stability test is more effective than the conventional postural stability test for assessing postural stability in patients with CAI remains unknown.

Therefore, this study aimed to analyze the differences in static postural stability, including conventional and modified static postural stability, dynamic postural stability, and muscle endurance between patients with CAI and healthy controls, and to investigate the relationship between static postural stability and muscle endurance, thus determining the reliability and usefulness of the modified static postural stability test in patients with CAI. We hypothesized that static and dynamic postural stability and muscle endurance were poorer in patients with CAI than in healthy controls, and that modified static postural stability may be significantly correlated with muscle endurance.

## Methods

### Study design and patient enrollment

The study was approved by the Institutional Review Board of the Korea University Anam Hospital (IRB No: 2019AN0397), and informed consent was obtained from all participants prior to the commencement of this study. All studies were conducted in accordance with the relevant guidelines and regulations.

Initially, 47 patients with CAI receiving rehabilitation from October 2019 were included in this study. All patients underwent plain stress radiography and physical examination and were evaluated by two orthopedic surgeons. Any disagreements were resolved by consensus. The inclusion criteria were patients with CAI with mechanical ankle instability due to lateral ankle sprain (the presence of a talar tilt > 9° and a difference between sides > 3° or 3 mm in anterior drawer)^8^ with a history of constant symptoms and repetitive sprains for 12 months prior to the study^20,21^. Patients with bilateral ankle sprains, deltoid ligament injuries, and acute ankle sprains were excluded. We also excluded patients with concomitant injuries causing knee and lower back pain as this would affect the postural stability test. In total, 26 healthy controls were selected from our database of volunteers with no history of sprains on either ankle for participation in this study. After excluding 21 patients, 26 patients with CAI and 26 healthy controls were enrolled in this study.

### Assessment of postural stability

**Figure 1:**
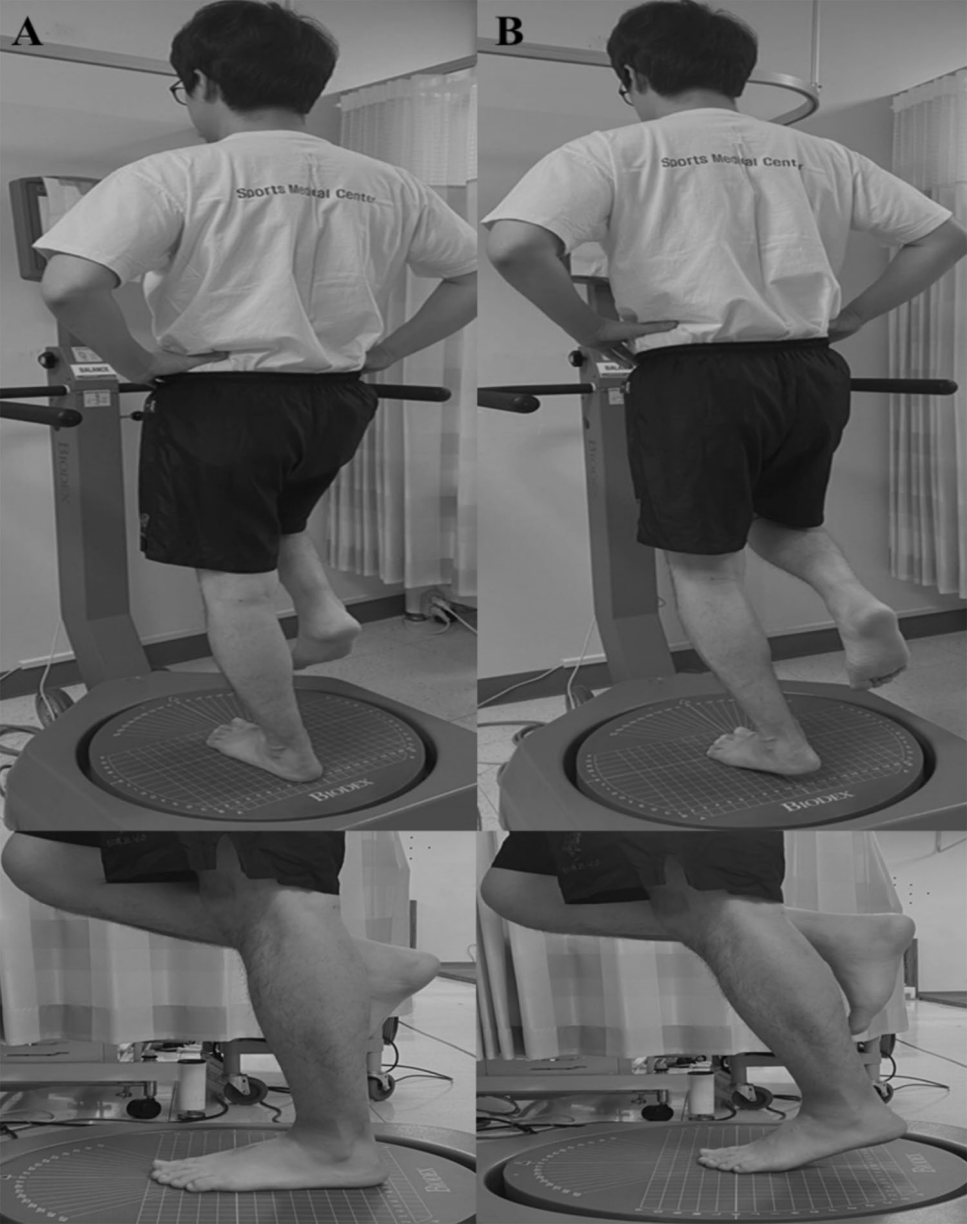
Static postural stability test position. The conventional static postural stability test (**a**) and modified static postural stability test (**b**).

The postural stability test was performed using the Biodex Stability System (BSS; Biodex Medical Systems, Inc., Shirley, NY, USA), which comprises static and dynamic postural stability tests. The static postural stability test uses a fixed platform, whereas the dynamic postural stability test uses a mobile platform. In the present study, static postural stability consisted of conventional static and modified static postural stability tests. The conventional static postural stability test (Fig. 1A) was conducted in a single-leg stance with the knee straight on a fixed platform^8^, whereas the modified static postural stability test (Fig. 1B) was conducted with the knee straight while the patient maintained a heel lift of approximately 2–3 cm on a fixed platform^22^. During the modified static postural stability test, one physical therapist continuously instructed the participants to maintain an approximately 2–3 cm heel lift using a stick ruler, and the test was terminated if the posture could not be maintained, or the proper heel lift was not performed. Dynamic postural stability was evaluated in a single-leg stance on a mobile platform ranging from level 12 (stable platform) to level 1 (unstable platform), with the stability level automatically declining every 1.66 s, with a 20° tilt and 360° platform rotation^8^. All participants were barefoot on the platform during the measurements, with the device screen covered to minimize visual compensation, wherein they performed two trials for 20 s each leg. A greater stability index (overall stability index [OSI], degree) is indicative of poor postural stability.

### Assessment of muscle endurance

The muscle endurance test (concentric-contraction mode) was evaluated in dorsiflexion and plantarflexion in a semi-seated position with 20° knee flexion using a quantified isokinetic device. Total work (J) was considered to represent muscle endurance and was defined as the sum of all torque curves during the 15 repetitions of dorsiflexion and plantarflexion at an angular velocity of 120°/s^9,23^.

### Sample size estimation and statistical analysis

Based on the results of previous studies on postural stability in patients with CAI^8,9^, a difference of > 0.5 in the OSI between the two groups was considered to be clinically significant. A priori power analysis was performed, with a significance level of 0.05, and a power of 0.8, to determine the sample size. According to the effect size (Cohen’s d: 1.056) calculated from the results of a pilot study involving 5 ankles in each group, 16 ankles in each group would be required to identify a clinically meaningful difference between the two groups. The power of this study was 0.825.

Student’s *t*-test was used to compare the differences in the conventional static and modified static postural stability, dynamic postural stability, and muscle endurance between the two groups. To determine whether a continuous variable followed a normal distribution, the Shapiro–Wilk test was used. Correlations between conventional static and modified static postural stability and muscle endurance were assessed using Pearson’s correlation coefficients. Regression analysis was performed using the significant variables of the Pearson’s correlation coefficients. Cohen’s d was calculated to examine the effect of statistical differences and was classified as weak (< 0.5), moderate (0.5–0.79), or large (> 0.8)^24^. Statistical analysis was performed using SPSS (SPSS for software version 21.0; IBM Inc., Chicago, IL, USA); P values < 0.05 were considered statistically significant.

## Results

Demographic data of the enrolled patients and healthy controls are summarized in Table 1; there were no significant differences between the two groups.

**Table 1:** Demographic data of the patients with CAI and healthy control subjects.

	CAI group	Healthy control group	*P*-value	Effect size
Sample size	26	26		
Sex (male/female)	17/9	20/6	0.341	
Age (years), mean (SD)	25 (6.9)	26 (5.9)	0.563	−0.077
Height (cm), mean (SD)	171 (7.0)	168 (9.1)	0.085	0.181
Weight (kg), mean (SD)	75 (14.7)	73 (12.9)	0.512	0.072
BMI (kg/m^2^), mean (SD)	25 (4.1)	26 (3.8)	0.770	−0.125
Sports and activity, n (low:high)	7/19	11/15	0.462	

*CAI* chronic ankle instability,; *SD* standard deviation.

### Reliability of the static postural stability test

To quantify the test–retest reliability of the conventional static and modified static postural stability, intraclass correlation coefficients (ICC) were calculated for two trials in each group performed by one physical therapist. The test–retest reliability of the conventional static and modified static postural stability tests was acceptable in the patients with CAI and healthy controls (ICC = 0.87 and ICC = 0.91 for the conventional static test; ICC = 0.80 and ICC = 0.86 for the modified static test, respectively).

### Postural stability test

In the modified static postural stability (single-leg heel-raise balance) and dynamic postural stability tests, the OSI was significantly higher in the affected ankles of patients with CAI than in healthy controls (modified static: 2.7 ± 0.9° vs. 1.7 ± 0.4°, *P* < 0.001, effect size [Cohen's d] = 1.43; dynamic: 2.8 ± 1.4° vs. 1.3 ± 0.7°, *P* < 0.001, effect size [Cohen's d] = 1.35). There were no significant differences between the two groups in terms of either the conventional static postural stability test or the unaffected ankles (*P* > 0.05, Table 2).

**Table 2:** Modified static postural stability, conventional static postural stability, dynamic postural stability, and muscle endurance in patients with CAI and healthy controls.

	Affected ankles			Unaffected ankles		
	CAI group	Healthy control group	*p*-value	CAI group	Healthy control group	*p*-value
Modified static postural stability, mean (SD)	2.7 (0.9)	1.7 (0.4)	**0.000**^**a**^	1.6 (0.5)	1.6 (0.5)	0.857
*MD, (95% CI)*	1.0, (0.6, 1.3)			0, (−0.2, 0.3)		
*Cohen’s d *	1.43			0		
Conventional static postural stability, mean (SD)	1.2 (0.3)	1.0 (0.4)	0.607	1.1 (0.4)	1.0 (0.4)	0.113
*MD, (95% CI)*	0.2, (0, 0.3)			0.1, (−0.1, 0.3)		
*Cohen’s d *	0.56			0.25		
Dynamic postural stability, mean (SD)	2.8 (1.4)	1.3 (0.7)	**0.000**^**a**^	1.4 (0.5)	1.2 (0.5)	0.168
*MD, (95% CI)*	1.5, (0.9, 2.1)			0.2, (0, 0.5)		
*Cohen’s d *	1.35			.39		
Dorsiflexion endurance, mean (SD)	93 (49.3)	110 (41.3)	0.198	99 (37)	115 (28.3)	0.086
*MD, (95% CI)*	−17, (−41.8, 8.9)			−16, (−34.3, 2.4)		
*Cohen’s d *	−0.37			−0.48		
Plantarflexion endurance, mean (SD)	235 (68.1)	280 (71)	**0.023**^**a**^	279 (134)	291 (80)	0.994
*MD, (95% CI)*	−45, (−83.9, −6.4)			−2, (−61.9, 61.4)		
*Cohen’s d *	−0.64			−0.10		

Bold means statistically significant between groups.

Postural stability (degree) and muscle endurance (J) are expressed as mean ± standard deviation at 120°/s.

*CAI* chronic ankle instability,; *MD* mean difference,; *CI* confidence interval,; *SD* standard deviation.

^a^Statistically significant.

### Muscle endurance test

Plantarflexion endurance was significantly lower in the affected ankles of the patients with CAI than in the healthy controls (235 ± 68.1 J vs. 280 ± 71 J, *P* = 0.023, effect size [Cohen's d] = -0.64), but there was no significant difference between the unaffected ankles and the healthy controls (*P* > 0.05, Table 2). The dorsiflexion endurance test showed no significant differences between the affected and unaffected ankles in the CAI group and healthy controls (*P* > 0.05, Table 2).

### Correlations between static postural stability and muscle endurance

**Figure 2:**
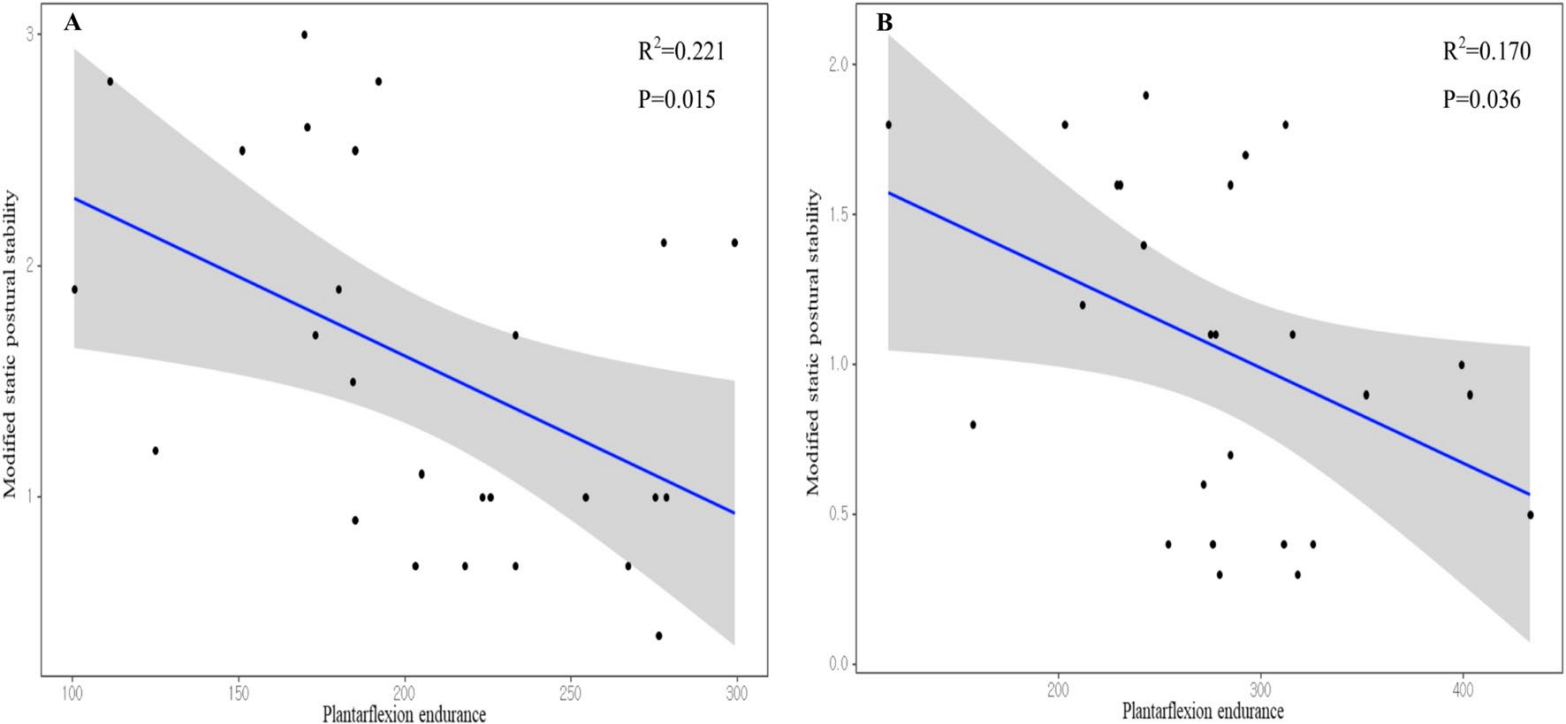
Plots show correlations between the modified static postural stability and plantarflexion endurance in CAI group (**a**) and healthy control group (**b**).

The correlations between conventional static and modified static postural stability and muscle endurance are shown in Table 3. There was a significant negative correlation between modified static postural stability and plantarflexion endurance (r = -0.470, *P* = 0.015), but not between the conventional static postural stability and dorsiflexion endurance (*P* > 0.05) in the affected ankles of patients with CAI. In healthy controls, there was a significant negative correlation between modified static postural stability and plantarflexion endurance (r = −0.413, *P* = 0.036 for the assessment of the affected ankles; r = −0.433, *P* = 0.027 for the assessment of the unaffected ankles), but not between conventional static postural stability and dorsiflexion endurance (*P* > 0.05). Regression analysis revealed that plantarflexion endurance was a significant risk factor for modified static postural stability in both the CAI group (R^2^ = 0.221, *P* = 0.015, Fig. 2A) and healthy controls (R^2^ = 0.170, *P* = 0.036, Fig. 2B).

**Table 3:** Correlations between modified static postural stability, conventional static postural stability, and muscle endurance.

Parameters	CAI group				Healthy control group			
	Affected ankles		Unaffected ankles		Affected ankles		Unaffected ankles	
	Modified static postural stability	Conventional static postural stability	Modified static postural stability	Conventional static postural stability	Modified static postural stability	Conventional static postural stability	Modified static postural stability	Conventional static postural stability
**Dorsiflexion endurance**								
PCC (r)	−0.051	−0.072	−0.131	−0.029	0.218	0.120	−0.132	0.124
P value	0.804	0.727	0.523	0.888	0.284	0.561	0.520	0.545
**Plantarflexion endurance**								
PCC (r)	−0.470	−0.354	−0.223	−0.045	−0.413	−0.107	−0.433	0.357
P value	**0.015**^**a**^	0.076	0.274	0.826	**0.036**^**a**^	0.603	**0.027**^**a**^	0.074

Bold means statistically significant between groups.

*PCC* Pearson’s correlation coefficient, *CAI* chronic ankle instability.

^a^Statistically significant.

## Discussion

The most important result of the present study was that the modified static postural stability (single-leg heel-raise balance), dynamic postural stability, and plantarflexion endurance significantly decreased in the affected ankles of the patients with CAI compared with healthy controls. Furthermore, modified static postural stability showed statistically significant correlations with plantarflexion endurance.

In previous studies, dynamic but not static postural stability was decreased in the affected ankles of patients with CAI^9,11,25^. In the present study, however, modified static postural stability was significantly decreased in the affected ankles of patients with CAI compared with healthy controls. This result may be explained by differences in the talocrural joint stability. The lateral ankle ligament complex, including the anterior talofibular ligament and the calcaneofibular ligament, plays a significant role in talocrural joint stability^1,2^, while talocrural joint instability due to lateral ankle ligament injury can affect postural stability^5,8,13^. The modified static postural stability test, which includes plantarflexion, was performed in the single-leg heel-raise position which decreases the stability of the talocrural joint and reinforces the stabilization of the talocrural joint by the ligamentous complex^26^. However, the conventional static postural stability test was performed in either neutral or dorsiflexion positions, both of which increase the stability of the talocrural joint and represent the locked position of the talocrural joint^26,27^. Although the modified static postural stability test assesses postural stability on a fixed platform, this test position may evaluate postural stability deficits owing to the increased instability of the talocrural joint in the affected ankles of patients with CAI. However, the static postural stability test was found to have a lower reliability^25,28,29^ and an inability to reflect the actual activities of daily living compared with the dynamic postural stability test^9,29^. Nevertheless, compared to the conventional static postural stability test, the modified static postural stability test may more effectively assess postural instability due to the decreased stability of the talocrural joint in patients with CAI.

In previous studies, the repeated heel-raise test was used to assess plantarflexion endurance^19,30^, which was reported to be lower in patients with CAI^15,31,32^, in line with our findings. In the present study, however, plantarflexion endurance was significantly correlated with the modified static postural stability test in patients with CAI. Although the reasons for this result are unclear, one possible reason may be the similar positioning of the ankle joint during testing. In the present study, both the plantarflexion endurance and modified static postural stability tests were performed with the ankle in plantar flexion, which may increase the micro-damage to the injured lateral ligamentous complex, thereby causing pain and resulting in the loss of muscle endurance and postural stability. Tabrizi et al.^33^. and Vitale and Fallat^34^ reported that the plantarflexion position may lead to an increased incidence of lateral ankle sprains. Moreover, previous studies on patients with CAI^35–37^ reported that ankle ligament deficits have been reported to lead to weaknesses in reflexive muscle contraction. Another potential reason for this result may be the impact of midfoot stability. The subtalar joint is in an inverted position in the plantar flexion position of the ankle joint, resulting in increased midfoot stability. Park et al.^15^. reported that midfoot stability may directly impact postural stability, which may indicate that decreased plantarflexion endurance affects the modified static postural stability test more significantly than it affects the other postural stability tests in patients with CAI. In the present study, statistically significant correlations between plantarflexion endurance and modified static postural stability were also demonstrated in healthy controls. Further investigation of patients with CAI without decreased plantarflexion endurance is necessary to assess the accuracy and efficacy of the modified static postural stability test and to clarify the results of the present study.

This study has several limitations. First, core muscle strength was not evaluated in this study, since Kibler et al.^38^. reported that core muscles play an important role in postural control. Second, we did not measure eccentric plantarflexion strength, although previous studies have reported that eccentric plantarflexion endurance significantly influences postural control^39,40^. However, Hubbard et al.^41^. reported that the concentric plantarflexion strength deficit was significantly greater in patients with CAI, which may also affect postural control. Therefore, the use of concentric and eccentric strengthening exercises for postural stability after rehabilitation in patients with CAI should be investigated. In conclusion, given its apparent reliability in assessing static postural stability and plantarflexion endurance, clinicians and therapists should consider using the single-leg heel-raise balance test to gauge improvements in postural stability and muscle endurance in patients with CAI before and after rehabilitation.

## Data Availability

All data generated or analyzed during the current study will not be disclosed due to the policy of the Korea University Anam Hospital Research Ethics Board.
